# Understanding Electrolytes and Interface Chemistry for Sustainable Nonaqueous Metal–CO_2_ Batteries

**DOI:** 10.1007/s40820-025-01801-5

**Published:** 2025-06-16

**Authors:** Bijiao He, Yunnian Ge, Fang Zhang, Huajun Tian, Yan Xin, Yong Lei, Yang Yang

**Affiliations:** 1 Beijing Laboratory of New Energy Storage Technology and Key Laboratory of Power Station Energy Transfer Conversion and System of Ministry of Education, School of Energy Power and Mechanical Engineering, North China Electric Power University, Beijing, 102206 People’s Republic of China; 2 Fachgebiet Angewandte Nanophysik, Institut Für Physik & ZMN MacroNano (ZIK), Technische Universität Ilmenau, 98693 Ilmenau, Germany; 3 NanoScience Technology Center, Department of Materials Science and Engineering, Renewable Energy and Chemical Transformation Cluster, Department of Chemistry, The Stephen W. Hawking Center for Microgravity Research and Education, University of Central Florida, Orlando, FL 32826 USA

**Keywords:** Nonaqueous metal–CO_2_ battery, Electrolytes and interface chemistry, Mechanism, Interface engineering, Solid electrolyte interface chemistry

## Abstract

Metal–carbon dioxide (CO_2_) batteries hold great promise for reducing greenhouse gas emissions and are regarded as one of the most promising energy storage techniques due to their efficiency advantages in CO_2_ recovery and conversion. Moreover, rechargeable nonaqueous metal–CO_2_ batteries have attracted much attention due to their high theoretical energy density. However, the stability issues of the electrode–electrolyte interfaces of nonaqueous metal–CO_2_ (lithium (Li)/sodium (Na)/potassium (K)–CO_2_) batteries have been troubling its development, and a large number of related research in the field of electrolytes have conducted in recent years. This review retraces the short but rapid research history of nonaqueous metal–CO_2_ batteries with a detailed electrochemical mechanism analysis. Then it focuses on the basic characteristics and design principles of electrolytes, summarizes the latest achievements of various types of electrolytes in a timely manner and deeply analyzes the construction strategies of stable electrode–electrolyte interfaces for metal–CO_2_ batteries. Finally, the key issues related to electrolytes and interface engineering are fully discussed and several potential directions for future research are proposed. This review enriches a comprehensive understanding of electrolytes and interface engineering toward the practical applications of next-generation metal–CO_2_ batteries.

## Highlights

- This review focuses on the design principles and basic characteristics of electrolytes, as well as how to construct a stable electrode–electrolyte interface. Perspectives on how electrolytes influence CO_2_ redox pathways are consolidated and proposed.
- The electrochemical reaction mechanism and interfacial evolution of nonaqueous metal–CO_2_ batteries in different electrolyte systems are highlighted.
- The electrode/electrolyte interface challenges encountered by nonaqueous metal–CO_2_ batteries are thoroughly discussed, along with corresponding optimization strategies.

## Graphical abstract

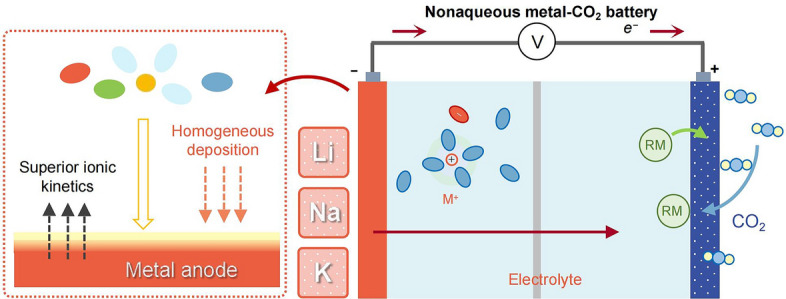

## Introduction

The energy dilemma caused by global warming and the depletion of fossil energy poses a serious challenge to the sustainable development of humankind [1–3]. The climate problem is largely attributable to the continued consumption of fossil fuels and the escalation of greenhouse gas emissions as a result of human activities [4]. Among the greenhouse gases known to have an impact on climate warming, carbon dioxide (CO_2_) poses the greatest threat [5, 6]. In recent years, researchers around the world have developed many new technologies to reduce CO_2_ emissions as well as to capture CO_2_ and convert it into usable energy and valuable chemical materials [7]. Electrochemical CO_2_ reduction provides an effective and sustainable method for capturing and converting CO_2_ [8–11]. However, the thermodynamically stable C=O bonds in the CO_2_ molecule and the multi-electron/proton transfer control on the catalyst surface make the energy conversion efficiency of some systems unsatisfactory. Metal–CO_2_ batteries (MCBs) allow direct electrochemical reduction of CO_2_, which improves the energy conversion efficiency [12, 13]. Although research on MCBs remains in their infancy, they demonstrate unique advantages in CO_2_ fixation, electrochemical conversion and integrated energy storage [14–16]. Upon capture, CO_2_ can be transformed into value-added chemicals, such as CO, methanol, formic acid, etc., through specifically designed catalysts or reaction pathways [17, 18]. The energy storage characteristics of MCBs enable their integration with renewable energy systems (e.g., wind and solar power), achieving “charge–discharge cycles” within power grids—surplus electricity drives CO_2_ reduction reactions for energy storage during charging, while discharging releases electrical energy while simultaneously fixing CO_2_, thereby contributing to carbon cycle balance. Furthermore, in specialized high-CO_2_ concentration environments (e.g., Martian atmosphere, or enclosed undersea spaces), MCBs show potential as self-powered energy solutions for detection equipment [19–21]. Their inherent capability to directly utilize environmental CO_2_ as active material makes them particularly promising for such application scenarios. This dual functionality of energy storage and CO_2_ utilization positions MCB technology as a prospective candidate for sustainable energy-carbon management systems.

An MCB uses a metal with negative electrode potential as the anode and CO_2_ as the cathode active material. The metal anodes that have been studied so far include lithium (Li), sodium (Na), potassium (K), zinc (Zn), aluminum (Al) and magnesium (Mg). Due to the different chemical activity of the metal anode, the water vapor and oxygen (O_2_) resistance of the MCBs is also different. Batteries using nonaqueous metals (Li/Na/K) can provide high energy density but are limited by their activity and are usually fitted with organic liquid solvents or solid-state electrolytes [22]. Zn/Al/Mg–CO_2_ batteries are compatible with water and are prepared under less stringent conditions. However, their low operating voltage and energy density also limit their practical application.

The electrolyte is the “blood” of the battery [23], affecting the main performance of the battery. Practical electrolytes should have high ionic conductivity, good thermal stability, chemical/electrochemical stability (no chemical reaction with the collector or active substance inside the battery and a wide electrochemical stability window (ESW)), environmentally friendly, low cost, and can be adjusted through manipulation of the solvation structure [24–27].

**Fig. 1:**
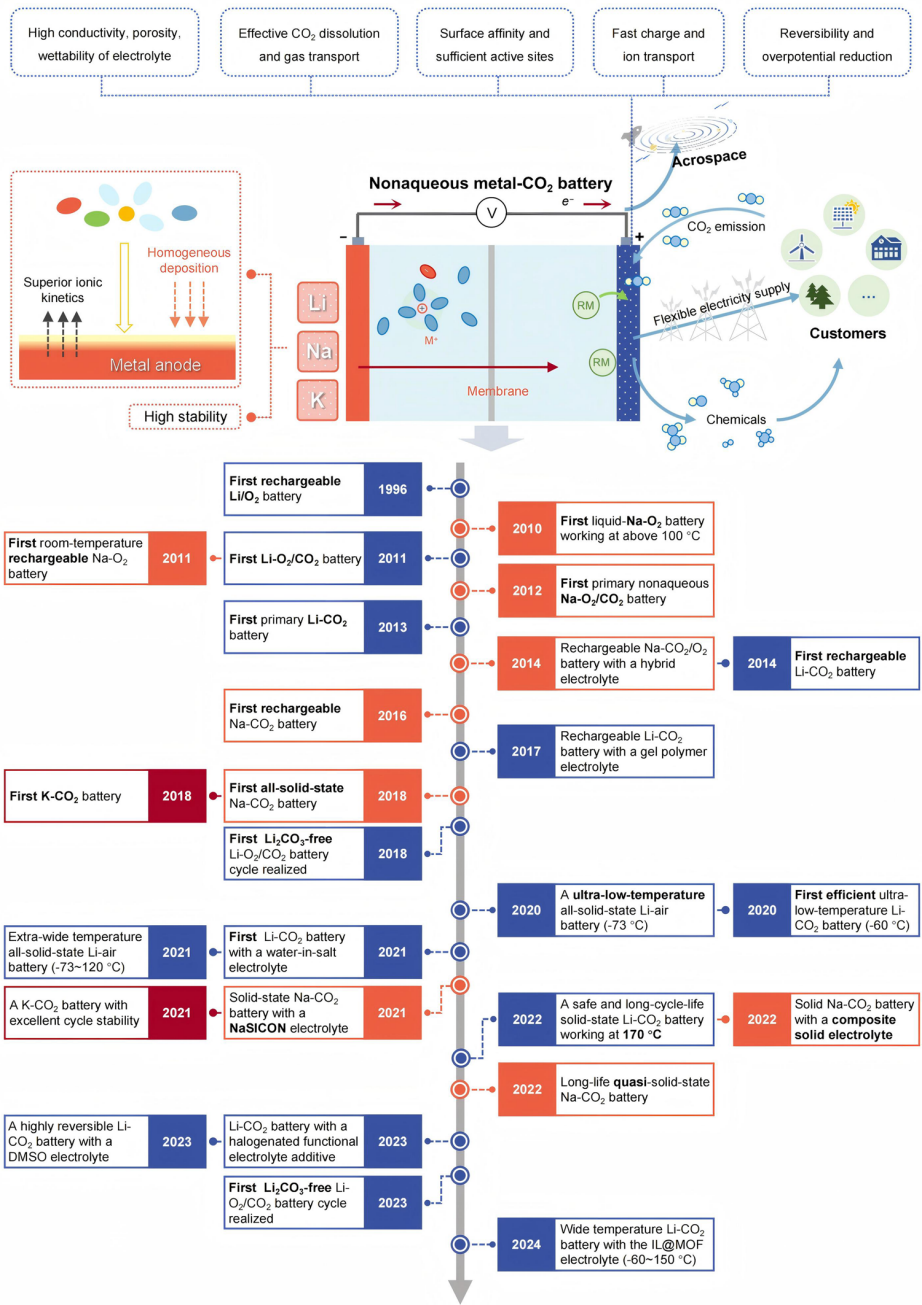
Schematic diagram and monumental developments of the nonaqueous MCBs and design principles of the battery device. The brief timeline starts with the metal–air batteries and mainly focuses on the development of electrolytes and interface engineering of nonaqueous MCBs

Although MCBs are still in the infancy, there is no doubt regarding their inherent advantages in energy storage and CO_2_ mitigation. Over the last decade, considerable efforts have been made to develop nonaqueous MCBs. The electrochemical performance has been progressively enhanced by developing novel cathodes and efficient catalysts, as well as designing the anode surface structures and regulating the electrolytes. The timeline of the origins of nonaqueous MCBs and research progress on the electrolytes is described in Fig. 1. MCBs were derived from metal–air batteries, a concept first proposed by Littauer et al. in 1976 [28]. Metal–air batteries utilize atmospheric O_2_ as a reactant, but CO_2_ and water vapor in the air are involved in the reaction to generate metal carbonates and metal hydroxides, which seriously damage the battery performance [29]. While investigating the effect of CO_2_, it was discovered that CO_2_ could be used as a reaction gas alone [30–32], resulting in MCBs were therefore investigated. Li–CO_2_ batteries were the first and most widely studied system. However, driven by the global lithium supply shortage issue, the more abundant elements, Na and K, were seen as alternatives to Li, and rechargeable beyond-Li(Na/K)–CO_2_ batteries entered a renaissance, being developed in 2016 and 2018 [32, 33], respectively. In early studies, nonaqueous organic electrolytes were conventionally employed for battery assembly. However, such semi-open battery structures inherently face risks of flammability and high-voltage decomposition. Solid-state electrolytes have emerged as a viable pathway to mitigate these challenges. Current research on solid-state nonaqueous MCBs remains in its nascent stage, with predominant focus on polymer electrolytes. In 2017, a Li–CO_2_ battery utilizing a gel polymer electrolyte was reported. By impregnating the polymer matrix with tetraglyme-based liquid electrolyte, the crystallization behavior of Li_2_CO_3_ discharge products was modulated, yielding substantially enhanced electrochemical performance compared to prior studies [34]. Subsequently, the first all-solid-state Na–CO_2_ battery was developed [35], followed by a variant incorporating an oxide-based solid state electrolyte [36]. The system demonstrated exceptional cyclability, achieving over 50 cycles at a specific capacity of 500 mAh g^−1^. Nevertheless, critical barriers persist for practical large-scale deployment of nonaqueous MCBs, including poor temperature tolerance (manifested as low ionic conductivity at low temperatures and electrolyte volatilization/decomposition at high temperatures). Consequently, wide-temperature-range nonaqueous MCBs have gradually emerged to address these operational limitations[37, 38]. The development of nonaqueous MCBs has made a qualitative leap, but there are still many challenges for nonaqueous MCBs, such as limited cycle life, low energy efficiency and poor rate performance [39–41]. Many eye-catching ideas have been developed in the areas of catalytic cathode materials, electrolytes and metal anodes.

Unlike other critical reviews, this paper first reviews the electrochemical mechanism of nonaqueous MCBs. Then, the design principles and basic characteristics of electrolytes are discussed with emphasis, combined with the determinants of designing high-performance nonaqueous MCBs. Furthermore, considering that an unstable interface can seriously degrade the safety and performance (especially the Coulombic efficiency and cycle life) of the battery, we also discuss how to construct a stable electrolyte/electrode interface. On this basis, we put forward several possible directions for future research. We hope that this review will provide a reliable understanding for designing high-performance metal–CO_2_ batteries.

## Configuration and Fundamental Mechanisms of MCBs

MCBs can be roughly divided into two categories: aqueous MCBs and nonaqueous MCBs. To understand nonaqueous MCBs, this section begins with an introduction to their electrochemical mechanisms and compares them with aqueous MCBs based on recent research reports. We discuss the CO_2_ reduction mechanism involving O_2_ participation, as well as the electrochemistry of MCBs.

### Discharge/Charge Mechanisms of Aqueous MCBs

Aqueous electrolytes are often paired with less reactive multivalent metals, offering advantages such as safety, low cost and low environmental pollution [42]. Generally, Al and Mg metals are not suitable for aqueous electrolytes because their low reduction potentials (− 1.66 and − 2.36 V, respectively, vs SHE) exceed the stable voltage range of H_2_O [43–46]. The nonaqueous AlCl_3_/[EMIm]Cl ionic liquid (IL) has become the preferred electrolyte for Al–CO_2_ batteries due to its excellent electrochemical performance [47, 48]. In rechargeable nonaqueous Mg–CO_2_ batteries, the introduction of moisture has been proven to alter the reversibility of reactions and the types of discharge products, resulting in long cycle life, high discharge voltage and capacity [49].

**Fig. 2:**
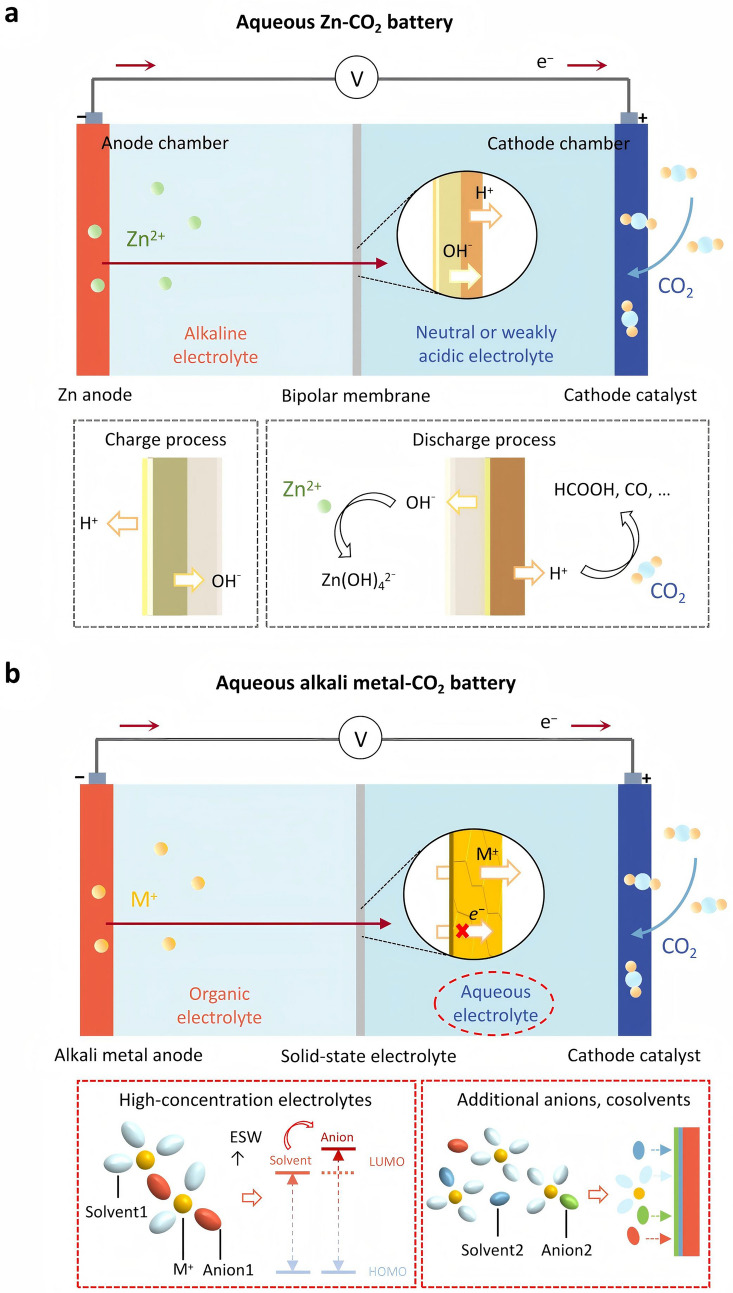
Schematic diagram of the **a** aqueous Zn–CO_2_ battery and **b** aqueous alkali metal–CO_2_ battery

Due to the low activity of Zn, there is no need to control the water and oxygen content in the environment, so aqueous electrolytes are chosen to assemble the battery. The working principle of the reversible Zn–CO_2_ battery is mainly based on the electrochemical reaction of the electrolyte. Zn is more effective in alkaline electrolytes, while CO_2_ will have side reactions with alkaline solutions, the Zn–CO_2_ battery device is divided into an anode chamber and a cathode chamber—one side is the zinc electrode and an alkaline electrolyte, and the other side is a catalyst and a neutral or weakly acidic electrolyte [50]. A bipolar membrane (BM) is used to maintain different pH values of the electrolytes on both sides (Fig. 2a). However, to meet the opposite charge-transfer requirements during charging and discharging, at least one pair of BMs in opposite directions must be set. When protons are sufficient, CO_2_ can controllably generate various chemicals, such as CO and HCOOH, based on the proton-coupled electron transfer mechanism [14, 41]. The Zn–CO_2_ battery utilizes this principle, combined with a highly selective catalyst cathode, to achieve a highly selective generation of CO [51] (Eq. 1).

1 $${\text{Zn}} + {\text{CO}}_{2} + 2{\text{H}}^{ + } + 4{\text{OH}}^{ - } \to {\text{Zn}}\left( {{\text{OH}}} \right)_{4}^{2 - } + {\text{CO}} + {\text{H}}_{2} {\text{O}}$$

In aqueous alkali metal–CO_2_ batteries, the discharge products formed by the reaction involving H_2_O have good solubility and do not clog the cathode pores like those in organic systems [52]. However, alkali metals can rapidly undergo side reactions with water, resulting in anode corrosion and having a significant negative impact on battery performance. Therefore, researchers have designed a hybrid electrolyte structure [53], which involves using a solid-state electrolyte as a separator to protect the anode, an organic liquid electrolyte on the anode side and an aqueous electrolyte on the catalyst cathode side (Fig. 2b). By leveraging the solubility advantage of the aqueous electrolyte discharge products and increasing the limited contact area between the discharge products and the catalyst cathode, the formation and decomposition of the discharge products are accelerated. However, the application of aqueous electrolytes is limited by their narrow ESW, as electrolysis occurs at a low voltage of 1.23 V, leading to hydrogen evolution reaction (HER) or oxygen evolution reaction (OER). These reactions shorten battery lifespan and inevitably reduce battery energy density [17, 54, 55], limiting practicality. To improve the electrochemical stability of aqueous electrolytes, one strategy is to alter the local chemical environment of water molecules to increase their inertness [56, 57]. This is primarily achieved by using salts with high solubility in water to prepare high-concentration electrolytes, such as lithium bis(trifluoromethanesulfonyl)imide (LiTFSI) and zinc chloride (ZnCl_2_) [50, 58]. As the salt concentration increases, the number of free solvent molecules decreases. Anions participate more in the solvation shell, enhancing the passivation ability of the electrolyte on the electrode, driving the transfer of the lowest unoccupied molecular orbital (LUMO) from the solvent to the anions, thus broadening the ESW [59–61].

However, the underlying mechanisms remain controversial, and high-concentration electrolytes tend to reduce conductivity and increase viscosity. Another strategy is to introduce additional anions, cosolvents, etc., to promote the growth of electrolyte interfaces similar to the solid electrolyte interface (SEI) on the electrodes, thereby kinetically inhibiting the electrolysis of electrolytes [62].

### Electrochemical Mechanisms of Nonaqueous MCBs

Although aqueous electrolytes generally exhibit superior charge-transfer capability compared to nonaqueous electrolytes under identical conditions due to their low viscosity and high ion dissociation degree [63]. Moreover, aqueous MCBs achieve long cycling stability by leveraging the high solubility of solid discharge products (e.g., metal carbonates) in water. Their inherent safety further renders them suitable for large-scale energy storage applications. However, their practical application is significantly constrained by inherent limitations, including the narrow ESW and poor low-temperature performance (the formation of ice crystals in aqueous electrolytes occurs below 0 °C) [64]. In contrast, nonaqueous MCBs achieve superior energy density, as organic electrolytes or ionic liquids enable a wide ESW [65], and maintain functionality under subzero temperatures.

**Fig. 3:**
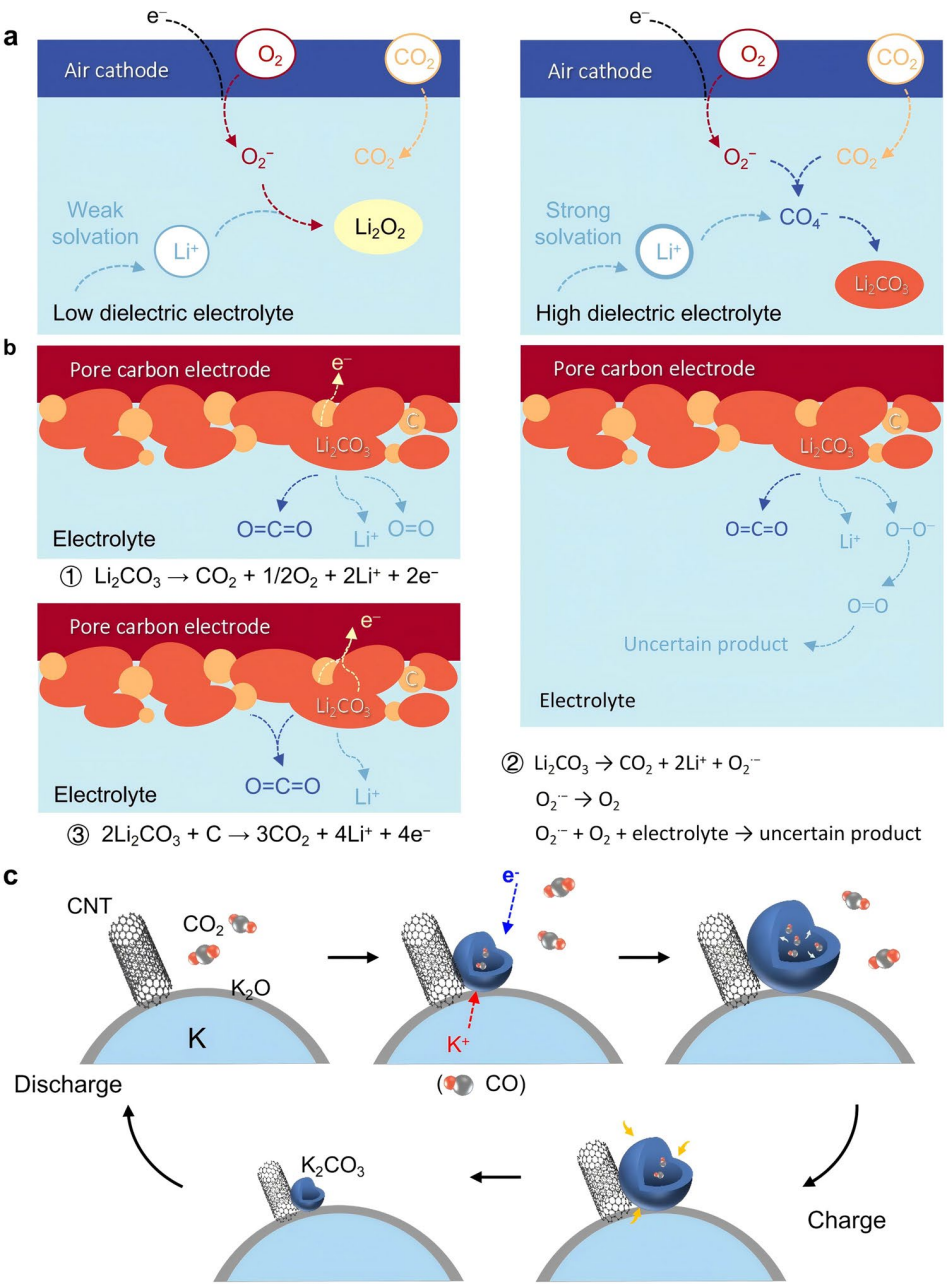
**a** The effect of the dielectric constant (DC) of the electrolyte solvent on the reaction process of a Li–O_2_/CO_2_ battery. **b** Possible reactions of the decomposition of Li_2_CO_3_. **c** Schematic representation of the growth and decomposition processes of the ball-like products in the K–CO_2_ battery. During discharging, the spherical structure of K_2_CO_3_ swells with the formation of CO gas. The sphere shrinks during charging, generating K and CO_2_

Before the advent of nonaqueous MCBs, researchers studied lithium-air batteries to understand the effect of ambient air during the reaction. At the cathode side, O_2_ accepts electrons from an external circuit and undergoes an oxygen reduction reaction (ORR), and the reduced O_2_ forms the superoxide anion radical ${\text{O}}_{2}^{\cdot -}$ in the organic liquid electrolyte [66]. The combination of ${\text{O}}_{2}^{\cdot -}$ with Li^+^ in the electrolyte leads to the final discharge products [67, 68]. Inspired by the fact that ${\text{O}}_{2}^{\cdot -}$ can be captured by CO_2_ to generate metal carbonates, 2011 witnessed the emergence of Li–O_2_/CO_2_ batteries [69]. Although CO_2_ is less concentrated in the environment, it is much more soluble in organic solvents than O_2_ (about 50 times more soluble) [69, 70]. The Li–O_2_/CO_2_ battery shows a higher discharge capacity as compared to the pure O_2_ as a reaction gas. The excellent electrochemical performance of the Li–O_2_/CO_2_ battery originated from the rapid consumption of ${\text{O}}_{2}^{\cdot -}$ by CO_2_ and the slow-filling characteristics of the final discharge product Li_2_CO_3_ in the cathode [69]. But at that time, the reaction mechanism of Li–O_2_/CO_2_ batteries was immature, and the source of the contribution to the discharge capacity was still debatable [71]. It should be noted that the electrolyte solvation effect can change the reaction pathway and final discharge products by altering the potential energy surface and controlling the formation of initial complex formation [72]. Quantum mechanical simulation verified that the discharge product tends to be Li_2_O_2_ in the low dielectric electrolyte, while the CO_2_ can be effectively electrochemically activated by the high dielectric electrolyte to promote the generation of more stable Li_2_CO_3_ (Fig. 3a).

In the early stages of research, CO_2_ was regarded as an “assistant” for O_2_ to increase the specific capacity and energy density of Li/Na–O_2_ batteries. Yet, in the case of Li–O_2_ batteries, CO_2_, while increasing the discharge capacity of the batteries, also spontaneously reacts with the main discharge product Li_2_O_2_ to form Li_2_CO_3_. Compared to the production of O_2_ from Li_2_O_2_ (~ 3–3.5 V) during battery charging [73], only at very high potentials (> 4 V) can Li_2_CO_3_ produce CO_2_. It does not seriously affect the discharge potential of Li–O_2_ batteries but reduces the efficiency of the discharge–charge cycles. Removing CO_2_ mixed in the air to prevent the formation of carbonate deposits is key to improving the stability of battery operation [74, 75]. In 2018, a Li_2_CO_3_-free Li–O_2_/CO_2_ battery was first realized [76]. The discharge products were successfully immobilized at the perovskite stage, which greatly reduced the charge overvoltage and the occurrence of the corresponding side reactions.

#### ***Li–CO***_***2***_*** Batteries***

The electrochemical mechanisms of nonaqueous MCBs are equally complex in the absence of O_2_ involved in the reaction. MCBs were first reported in 2013 by Archer et al. [31] who developed a high-temperature primary nonaqueous Li–CO_2_ battery based on an activated carbon cathode. The hypothesis of electrochemical reactions during discharging as in Eq. 2 was proposed, based on $E=-\Delta G/zF$, with $\Delta G$, $z$, and $F$ representing the change in Gibbs free energy, the number of electrons transferred per mole of product and the Faraday constant, respectively. However, it was found that at the high temperature, the actual discharge potential was higher than the theoretical discharge potential, which violated Tafel’s theory [12, 31]. The gas phase composition of the cell was further analyzed by differential electrochemical mass spectrometry (DEMS), and it was found that carbon monoxide (CO) as an intermediate product undergoes disproportionation (Eq. 3). Finally, Li_2_CO_3_ was deduced to be the main discharge product. The reaction process is shown in Eq. 4.

2 $$2{\text{Li}} + 2{\text{CO}}_{2} \to {\text{Li}}_{2} {\text{CO}}_{3} + {\text{CO}}$$

3 $$2{\text{CO}} \to {\text{CO}}_{2} + {\text{C}}$$

4 $$3{\text{CO}}_{2} + 4{\text{Li}} \to 2{\text{Li}}_{2} {\text{CO}}_{3} + {\text{C}}$$

CO_2_ would form the intermediate oxalate ions (${\text{C}}_{2}{\text{O}}_{4}^{2-}$) via a two-electron reduction reaction upon discharging, which in turn would be converted to stable oxalate with metal ions (Eqs. 5 and 6). Reversible electrochemistry can be achieved by selecting a suitable electrode/catalyst that converts CO_2_ to ${\text{C}}_{2}{\text{O}}_{4}^{2-}$ and oxidizes oxalate to CO_2_ with 100% selectivity [70, 77, 78]. Moreover, the conversion reaction does not involve toxic carbon CO and peroxides that can cause explosive combustion. Environmentally friendly and stable oxalates are good choices. However, the unstable Li_2_C_2_O_4_ is thermodynamically easier to decompose than Li_2_CO_3_, thus realizing the low overpotential of the battery [78–80], as shown in Eqs. 7 and 8:

5 $$2{\text{CO}}_{2} + 2{\text{e}}^{ - } \to {\text{C}}_{2} {\text{O}}_{4}^{2 - }$$

6 $$2{\text{Li}}^{ + } + {\text{C}}_{2} {\text{O}}_{4}^{2 - } \to {\text{Li}}_{2} {\text{C}}_{2} {\text{O}}_{4}$$

7 $${\text{Li}}_{2} {\text{C}}_{2} {\text{O}}_{4} \to 2{\text{CO}}_{2} + 2{\text{Li}}^{ + } + 2{\text{e}}^{ - } ,\,{\text{E}}^{0} = 3.01{\text{ V vs Li}}/{\text{Li}}^{ + }$$

8 $$2{\text{Li}}_{2} {\text{CO}}_{3} \to 2{\text{CO}}_{2} + {\text{O}}_{2} + 4{\text{Li}}^{ + } + 4{\text{e}}^{ - } ,\,{\text{E}}^{0} = 3.82{\text{ V vs Li}}/{\text{Li}}^{ + }$$

The unstable ${\text{C}}_{2}{\text{O}}_{4}^{2-}$ was hypothesized to undergo a two-step disproportionation [81] to form the more stable ${\text{CO}}_{3}^{2-}$ and C (Eqs. 9 and 10), followed by the formation of stable Li_2_CO_3_ (Eq. 11). The evolution of the reaction products was also proved by using in situ techniques [80]. It was also found that a new discharge plateau occurs at lower levels of potential drop during discharging, which can be explained by Eq. 12. The eventual transformation of Li_2_O to Li_2_CO_3_ is attributed to higher polarization potentials and changes in local CO_2_ concentration. With ruthenium (Ru) as the cathode catalyst, Ru particles can catalyze the reversible reaction of Li_2_CO_3_ with C [40] (Eq. 13), whereas, in the absence of Ru particles catalyzing the reaction, Li_2_CO_3_ tends to self-decompose at a high potential, resulting in the irreversible deposition of discharge by-products (Eq. 14):

9 $${\text{C}}_{2} {\text{O}}_{4}^{2 - } \to {\text{CO}}_{2} + {\text{CO}}_{2}^{2 - }$$

10 $${\text{C}}_{2} {\text{O}}_{4}^{2 - } + {\text{CO}}_{2}^{2 - } \to 2{\text{CO}}_{3}^{2 - } + {\text{C}}$$

11 $${\text{CO}}_{3}^{2 - } + 2{\text{Li}}^{ + } \to {\text{Li}}_{2} {\text{CO}}_{3}$$

12 $${\text{CO}}_{2} + 4{\text{Li}}^{ + } + 4{\text{e}}^{ - } \to 2{\text{Li}}_{2} {\text{O}} + {\text{C}}$$

13 $$3{\text{CO}}_{2} + 4{\text{Li}}^{ + } + 4{\text{e}}^{ - } \to 2{\text{Li}}_{2} {\text{CO}}_{3} + {\text{C}}$$

14 $${\text{Li}}_{2} {\text{CO}}_{3} \to 2{\text{Li}}^{ + } + {\text{CO}}_{2} + {\text{e}}^{ - } + {\text{O}}_{2}^{\cdot - }$$

Catalysts with different electrokinetic characteristics exhibit different electrochemical reaction routes, influenced by a variety of factors such as structure, composition and crystallinity [82]. Moreover, the electrocatalytic selection largely determines the final reduced species, and Li_2_CO_3_ with high structural stability is generally considered to be the final discharge product in the electrochemical process of Li–CO_2_ batteries.

There are three possible pathways for the electrochemical precipitation mechanisms of CO_2_ during charging [74], as shown in Fig. 3b. Pathway 1 is a direct decomposition reaction of Li_2_CO_3_. Pathway 2 is a complex process with multiple steps, and it describes a charging process with generated superoxide radicals (${\text{O}}_{2}^{\cdot -}$). ${\text{O}}_{2}^{\cdot -}$ is highly reactive and easily converts to O_2_, promoting the formation of a series of by-products by directly attacking the electrolyte solvent. Pathways 1 and 2 belong to the self-decomposition reactions of Li_2_CO_3_, and the generated carbon species during the discharge process are not involved in the reaction, so it is not possible to make the Li–CO_2_ battery carry out a reversible cycle [39]. The accumulation of the products will also lead to the deterioration of electrochemical performance. In pathway 3, Li_2_CO_3_ reacts with carbon species that may originate from the discharge reaction or on the cathode, and the design of suitable electrolytes and catalysts can realize the reversibility of the battery and reduce the battery charge voltage [82, 83].

#### ***Na–CO***_***2***_*** Batteries and K–CO***_***2***_*** Batteries***

The development of Li–CO_2_ batteries has been hampered by the global scarcity of lithium resources. Yet, the greater plenty of sodium and potassium in the same main group in the earth’s crust provides an opportunity for electrochemical applications in place of lithium. Chen et al. [32] reported a rechargeable room-temperature Na–CO_2_ battery. Its battery structure is similar to a Li–CO_2_ battery. The reversible electrochemical reaction of Eq. 15 was demonstrated by various characterizations such as in situ Raman spectroscopy and CO_2_ evolution tests:

15 $$3{\text{CO}}_{2} + 4{\text{Na}} \leftrightarrow 2{\text{Na}}_{2} {\text{CO}}_{3} + {\text{C}}$$

Like Li_2_CO_3_, Na_2_CO_3_ exhibits strong structural stability, which requires a high charge potential to drive its decomposition, leading to poor cycling stability of the batteries [84, 85]. To reduce the overpotential for Na_2_CO_3_ decomposition and to speed up the charge-transfer kinetics, the structure and composition of the cathode materials, as well as the cell configuration, need to be rationally designed [86]. Selecting the single platinum atom on nitrogen (N)-doped carbon nanotube (Pt@NCNT) as the cathode, during the discharge process, Na_2_CO_3_ spheres were formed on the surface of Pt@NCNT and the discharge reaction was as described in Eq. 16. During charging, Na_2_CO_3_ spheres decompose on the surface of CNTs into metallic sodium and CO_2_ as well as de-embedding of sodium ions in CNTs.

16 $$3{\text{CO}}_{2} + 4{\text{Na}}^{ + } + 4{\text{e}}^{ - } \leftrightarrow 2{\text{Na}}_{2} {\text{CO}}_{3} + {\text{C}}$$

With the low price of potassium salts and the plenty of potassium in nature, K–CO_2_ batteries are also expected to be one of the next-generation alternative systems for energy storage. The lower standard potential of K^+^/K (− 2.93 V vs SHE) compared to the Na^+^/Na electric pair (− 2.71 V vs. SHE) suggests that K–CO_2_ batteries have the potential for higher voltage output [87]. Compared with Li–CO_2_ and Na–CO_2_ batteries, there have been fewer reports on K–CO_2_ batteries. Zhang and co-workers [33] used the aberration-corrected environmental transmission electron microscope (AC-ETEM) to observe in situ the discharge/charge process of K–CO_2_ batteries to explore their electrochemical mechanisms (Fig. 3c). During the discharge process, K reacted with CO_2_ to form K_2_CO_3_ and CO (Eq. 17), which formed a large number of nanobubbles in the cathode, and the production of CO caused K_2_CO_3_ to expand into hollow structures. During the charging process, as the carbon in the electrode was consumed, K_2_CO_3_ decomposed into K and CO_2_ (Eq. 18), and the K_2_CO_3_ hollow spheres contracted during the charging process. It was also observed that the carbon nanotubes (CNTs) became thinner, indicating that the CNTs were consumed. This study provides a basic understanding of K–CO_2_ batteries, but as shown in Eqs. 18 and 19, the CO_2_ gas produced during discharging is not consumed during subsequent charging, implying that the batteries need to consider gas release for practical applications. The construction of a continuous dense SEI membrane on the surface of active potassium metal can inhibit the side reactions [88, 89] between the electrolyte and anode.

17 $$2{\text{CO}}_{2} + 2{\text{K}} \to {\text{K}}_{2} {\text{CO}}_{3} + {\text{CO}}$$

18 $$2{\text{K}}_{2} {\text{CO}}_{3} + {\text{C}} \to 4{\text{K}} + 3{\text{CO}}_{2}$$

19 $$3{\text{CO}}_{2} + 4{\text{K}} \leftrightarrow 2{\text{K}}_{2} {\text{CO}}_{3} + {\text{C}}$$

Besides the advantages of cost and high-voltage output, K^+^ has a weaker Lewis acidity than Li^+^ and Na^+^ [33], meaning that it migrates faster inside the electrolyte and at the electrode–electrolyte interface. Yet, the high reducibility of potassium metal also leads to the quite unstable electrochemical performance of K–CO_2_ batteries. The design of K–CO_2_ energy storage devices and the dissection of the reaction mechanism of K–CO_2_ batteries still need to be further investigated.

## Electrolyte Optimization Strategies of Nonaqueous MCBs and Interface Chemistry for Metal Anodes

### Optimization Strategies and Advances of Electrolytes

The electrolyte in MCBs not only affects the performance and efficiency of the battery but also determines the reaction mechanism and stability. Due to the high activity of alkali metals, nonaqueous electrolytes are employed, including liquid, quasi-solid-state or solid-state electrolytes.

Liquid electrolytes include organic liquid electrolytes and ionic liquids (ILs, molten salts) [62]. Liquid electrolyte solvents in nonaqueous MCBs are mainly organic solvents with high dielectric constants and low viscosities [90–93], which are conducive to the dissolution and ion migration. Compared to water, organic solvents exhibit a wider ESW, and they can form a stable passivation layer on the electrode surface. However, some side reactions in organic liquid electrolytes hinder the further improvement of battery performance. ILs have attracted attention due to their chemical stability, nonvolatility and relatively wide potential window. They exhibit excellent performance in CO_2_ capture and separation due to their unique properties and molecular structures [94]. In liquid systems, membranes are required to prevent battery short circuits. The membranes feature a porous structure. However, these pores are typically large, allowing electrolytes and ions to pass through while also facilitating the shuttle of gases [95], which can lead to side reactions with the anode. In contrast, solid-state electrolytes possess excellent denseness, which not only restricts the movement of liquid molecules but also effectively blocks the shuttle of CO_2_.

Quasi-solid-state electrolytes (QSEs) are gel-like substances formed from a polymer matrix with a liquid electrolyte or IL, also known as gel polymer electrolytes (GPEs), which combine the mechanical properties and high ionic conductivity with leakage resistance. Solid electrolytes include inorganic solid electrolytes (ISEs) and polymer solid electrolytes (PSEs). However, due to inherent limitations in both types, composite solid electrolytes (CSEs)—combining ISEs and PSEs—are favored to leverage the advantages of each. CSEs have high ionic conductivity, wide ESW, good mechanical properties and excellent flexibility [96]. Quasi-solid/solid-state electrolytes provide an opportunity to address the flammability and volatility of liquid solvents [79]. However, poor room-temperature ionic conductivity and high impedance at the electrode–electrolyte interface are common problems of quasi-solid/solid-state electrolytes at present [79]. The optimization of electrolytes and interface engineering needs to be studied in depth.

#### Discharge and Charge Plateau

Electrolytes can participate in redox reactions of CO_2_ by releasing soluble catalytically active substances. Redox mediators (RMs) are soluble molecules with reversible redox couples that act as intermediate charge carriers in redox reactions. It helps facilitate charge transfer between the two reactants and lowers the energy barrier required for final product formation. Efficient RMs should have some important characteristics [97–99]. First, the redox potential of RMs should be slightly higher than the thermodynamic potential of Li_2_CO_3_ decomposition, which helps to cut the overpotential of nonaqueous MCBs. Second, RMs should have high chemical/electrochemical stability to ensure that they do not react with other substances involved in the reaction process. Third, the RMs should have enough solubility in the solvent to ease the complete decomposition of the discharge products.

**Fig. 4:**
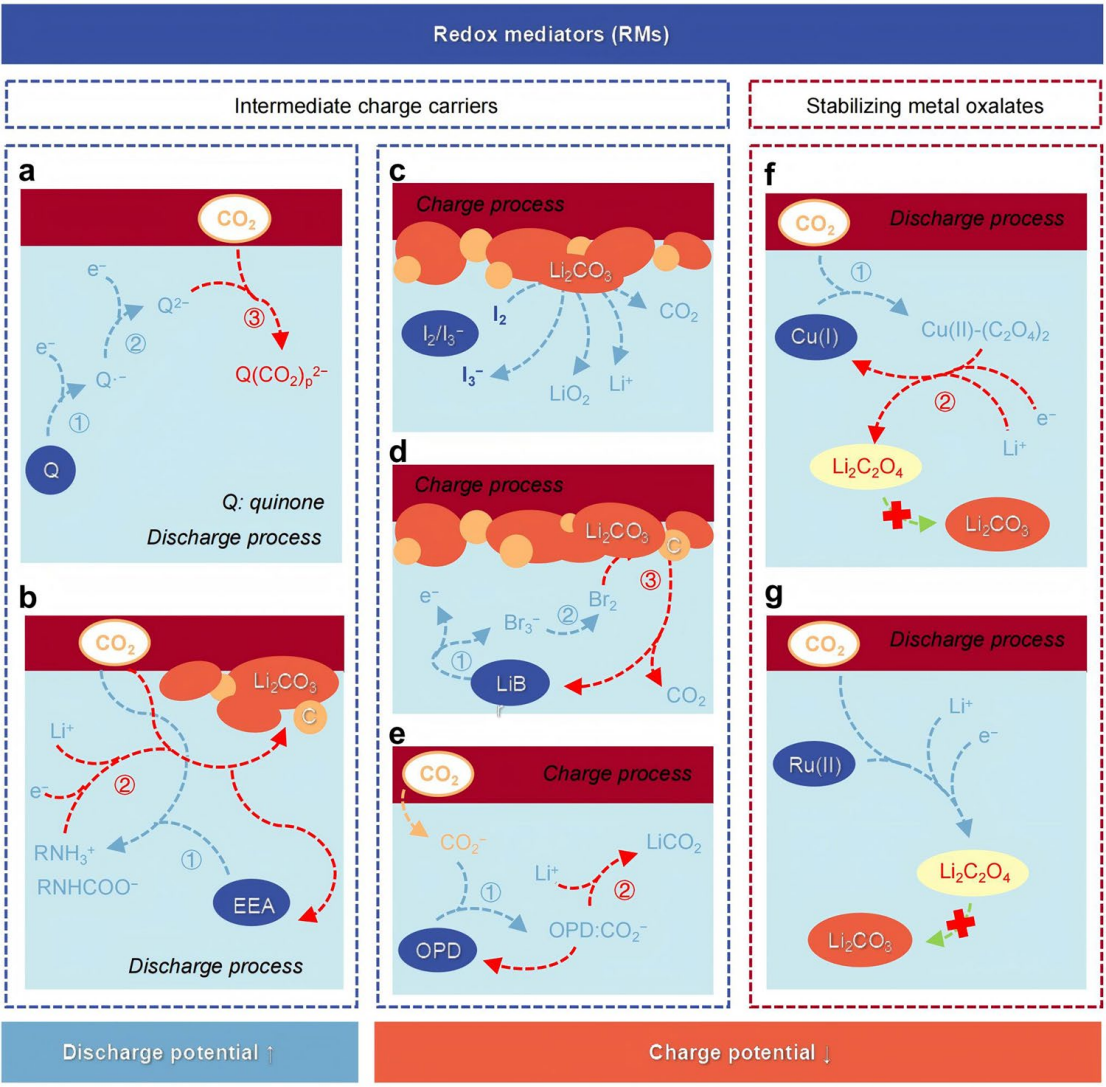
Pathways and examples of redox mediators (RMs) in battery charging and discharging. RMs act as intermediate charge carriers in redox reactions, facilitating charge transfer between reactants, **a, b** increasing the discharge plateau, and **c, d, e** decreasing the charging overpotential. **f, g** RMs stabilize metal oxalate products and contribute to the reversible cycling of cells

RMs can be used to increase discharge plateau by leveraging the unique chemical binding ability between RMs and CO_2_ [100, 101] (Fig. 4a, b). The strategy of immobilizing solid RMs, such as quinones (Qs) organic and 2-ethoxyethylamine (EEA)-CO_2_ adduct, on the cathode effectively avoids the issues of shuttle consumption and sluggish kinetics while retaining the functionality of soluble RMs [92, 101–105].

Adding a suitable RM to the electrolyte, acting as an intermediate charge carrier (Fig. 4c-e), can also help to reduce the charge potential [106–109]. When a small amount of iodine is added to the 1 M LiTFSI/trimethylphosphate (TMP) electrolyte, complexation of the iodine molecules with the solvent alters the oxidizing ability of the iodine [110]. The ${I}_{2}$/${I}_{3}^{-}$ mediator in the electrolyte promotes the decomposition of Li_2_CO_3_ at low charge voltages. Br_2_ can chemically oxidize the discharge products Li_2_CO_3_ and C [74, 111], while leaving ${\text{Br}}_{3}^{-}$ as a reduction product. The charge voltage of the battery with added LiBr was reduced from 4.5 to 4.0 V. Amine-based o-phenylenediamine (OPD) can form $\text{OPD}:{\text{LiCO}}_{2}$, a stable adduct, with CO_2_ intermediates [112], increasing the solubility of discharge products and promoting the liquid-phase growth of the discharge products, thereby enhancing the reaction kinetics and lowering the charge voltage.

RMs do not change the basic redox reactions of nonaqueous MCBs, but they can affect the charge potential of the batteries by altering specific reaction pathways and promoting the decomposition of discharge products. Besides, RMs can also reduce the charge potential by participating in the electrochemical reduction process to form discharge intermediate products, Li_2_C_2_O_4_ (Fig. 4f, g). When the soluble binuclear copper(I) complex was added as a liquid catalyst in the electrolyte, it first chemically reacted with CO_2_ to form Cu(II)-oxalate adducts, which then electrochemically reduced to form metal oxalate products and pristine Cu(I)RM [19]. This electrochemical process greatly increases the output voltage of the cell (> 3.0 V) while keeping the charge plateau relatively low (3.99 V) and also avoids aggressive intermediates from Li_2_CO_3_ decomposition. The Ru(II) centers of the complex tris(2,2’-bipyridyl)-dichloro-ruthenium(II) (Ru(bpy)_3_Cl_2_) stabilize the discharge intermediates by interacting with dissolved CO_2_ molecules, retarding their conversion to carbonates to reduce the charge voltage [113].

#### Ionic Conductivity

The ionic conductivity of an electrolyte is closely related to the concentration and migration rate of active ions. Therefore, the concentration of the electrolyte salt and the choice of the solvent are critical. Excellent intrinsic properties lead to the widespread use of TEGDME as an electrolyte solvent for nonaqueous MCBs [114–118]. Experiments have shown that TEGDME exhibits a peak in conductivity with LiTFSI concentration, which occurs at a Li^+^ concentration of 1 M (2.72 mS cm^−1^), as well as a low level of viscosity (< 10 centipoise, 25 °C) [119]. These properties enhance the availability of Li^+^ across the threshold required to support its activation. In 1 M LiTFSI/DMSO electrolyte, only 1 out of 12 DMSO molecules can dissolve lithium ions, while the rest corrode the anode and volatilize slowly. When the concentration of LiTFSI is increased to 4 M, these issues are fully ameliorated [120].

**Fig. 5:**
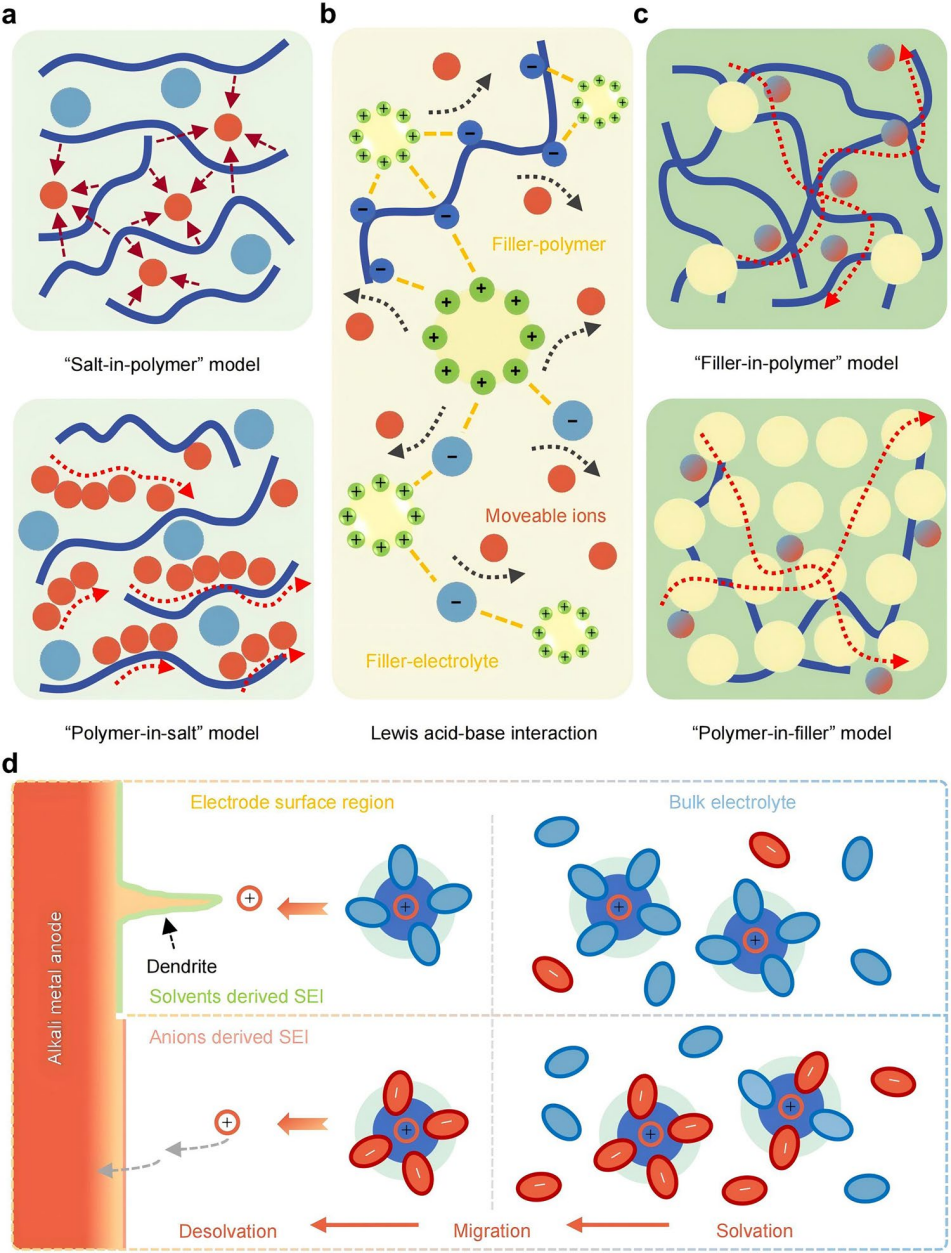
Schematic illustration of ion transport processes and solvation structures. **a** Ion transport pathways in polymer solid electrolytes. **b** Lewis acid–base interaction in composite solid electrolytes caused by the Lewis acid-type fillers. **c** Ion transport pathways in composite solid electrolytes. **d** The inducing effects of different solvation structures on SEI

QSEs have a high advantage in ionic conductivity due to the filling of liquid electrolytes, even approaching that of liquid electrolytes [1, 39]. The compact electrolyte structure mitigates the dissolution of CO_2_ in the electrolyte, while the liquid electrolyte concentrates the CO_2_ at the cathode interface. Such a structure reduces the contact of the metal anode with CO_2_. Although solid-state electrolytes can avoid some defects of liquid electrolytes, the ionic conductivity of solid-state electrolytes, especially PSEs, is usually lower than that of liquid electrolytes. Adding appropriate additives to PSEs is a feasible measure to improve ionic conductivity [100, 121]. On the one hand, ion migration in polymers is determined by the segmental mobility of the polymer chains, structural diffusion and ion hopping. The disordered nature of the polymer enhances the ordered motion of the polymer chains, allowing ions to move faster above the polymer glass transition temperature (${T}_{\text{g}}$) than ordered phases (crystalline phases) [122]. Therefore, high ionic conductivity of the PSEs can be achieved by creating a permeable network rich in amorphous domains. Decoupling ionic transport from the polymer chain through additives like plasticizers, metal oxides and ILs may be an effective strategy [123]. On the other hand, as Lewis bases, many polymer matrices strongly interact with metal ions (Lewis acids), thereby restricting cation mobility and lowering electrical conductivity (Fig. 5a) [124–126]. The addition of Lewis acid-type nanoparticles competes with metal cations for interaction with the Lewis base-type polymer matrix, releasing more free metal cations for conduction [131]. In addition, the Lewis acid–base interaction between the filler and the electrolyte salt promotes the dissociation of the electrolyte salt, which benefits ion transport and increases the concentration of moveable ions (Fig. 5b) [128].

The ionic conductivity of ISEs is better than that of PSEs, but they are criticized for their mechanical properties. Adding plastic crystalline materials, such as succinitronil (SN), can bring amorphism and flexibility. Also, the CSE is an improvement solution. ISEs usually possess one or several ion diffusion channels and a suitable ion concentration/vacancy ratio, which enables ions to move quickly in the crystal structure framework and achieve high ionic conductivity [129, 130]. For CSEs filled with inorganic fillers, on the one hand, inorganic particles act as the crosslinking center, which can reduce the crystallinity of the polymer and increase the ionic mobility, thus obtaining higher ionic conductivity (Fig. 5c). On the other hand, through the Lewis acid–base interaction, a uniformly distributed space charge layer is formed, which enhances the dissociation of the matrix and fixes the anions [131]. We introduce a series of engineered electrolyte formulations optimized for nonaqueous MCB systems, with their intrinsic physicochemical characteristics and corresponding electrochemical performance metrics systematically compiled in Table 1.

**Table 1:** Some typical achievements for electrolytes of MCBs in terms of functional materials and electrochemical properties

Electrolyte	Cathode material	Electrochemical window	The ionic conductivity, temperature	Full discharge capacity, current density	Voltage gap, applied current	Cyclability	References
*Li–CO*_*2*_* batteries*							
LiPF_6_-LiTFSI/TEGDME	Mo_3_N_2_	–	–	12.6 mAh cm^−1^, 20 μA cm^−1^	1.305 V, 200 μA cm^−1^	272 cycles with the cut-off capacity of 100 μAh cm^−2^ at 20 μA cm^−1^ 884 cycles with the cut-off capacity of 100 μAh cm^−2^ at 100 μA cm^−1^	[132]
Fc/LiTFSI in TEGDME	CNTs	–	–	15,900 mAh g^−1^, 100 mA g^−1^	–	137 cycles with the cut-off capacity of 500 mAh g^−1^ at 100 mA g^−1^	[133]
B3BPE/LiTFSI in TEGDME	Super P	–	–	13.9 mAh cm^−1^, 0.1 mA cm^−1^	1.36 V, 0.1 mA cm^−1^	120 cycles with the cut-off capacity of 0.2 mAh cm^−2^ at 0.1 mA cm^−1^	[134]
LiNO_3_-LiTFSI/[EMIm]^+^[BF_4_]/in DMSO	CoFe_2_O_4_/MWCNTs	5 V	~ 85 mS cm^−1^, RT	31,346.3 mAh g^−1^, 500 mA g^−1^	1.9 V, 500 mA g^−1^	100 cycles with the cut-off capacity of 1000 mAh g^−1^ at 500 mA g^−1^	[135]
LiFSI-KFSI	Ru@Super P	4.5 V	2.99 mS cm^−1^, RT	9503 mAh g^−1^, 100 mA g^−1^	1.15 V, 100 mA g^−1^	10 cycles with the cut-off capacity of 1000 mAh g^−1^ at 500 mA g^−1^	[136]
Binuclear CoPc	Novel pencil trace	4.5 V	0.86 mS cm^−1^, RT	27,196 mAh g^−1^, 100 mA g^−1^ 20,878 mAh g^−1^, 200 mA g^−1^ 15,786 mAh g^−1^, 500 mA g^−1^	1.14 V, 200 mA g^−1^	120 cycles with the cut-off capacity of 1000 mAh g^−1^ at 200 mA g^−1^	[100]
IL@MOF	CNTs-IL@MOF	4.71 V	1.03 mS cm^−1^, RT	13,978 mAh g^−1^, 50 mA g^−1^	–	441 cycles with the cut-off capacity of 1000 mAh g^−1^ at 500 mA g^−1^	[38]
Quasi-solid polymer electrolyte	Cellulose carbon aerogel-supported Ru nanoparticles	5.27 V	1.98 mS cm^−1^, RT	2.91 mAh cm^−2^, 50 μA cm^−2^	1.05 V, 50 μA cm^−2^	295 cycles with the cut-off capacity of 100 μAh cm^−2^ at 50 μA cm^−2^ 81 cycles with the cut-off capacity of 250 μAh cm^−2^ at 50 μA cm^−2^	[137]
PVDF-HFP-Li_6.5_La_3_Zr_1.5_Ta_0.5_O_12_-SN/LiTFSI	Commercial RuO_2_	4.7 V	1.11 mS cm^−1^, RT	–	–	34 cycles with the cut-off capacity of 500 mAh g^−1^ at 200 mA g^−1^	[96]
Polymer electrolyte	CNTs	5 V	0.36 mS cm^−1^, RT	12,000 mAh g^−1^, 100 mA g^−1^	~ 1.65 V, 100 mA g^−1^	100 cycles with the cut-off capacity of 1000 mAh g^−1^ at 100 mA g^−1^	[138]
Gel polymer electrolyte	CNTs	4.5 V	0.5 mS cm^−1^, RT	8536 mAh g^−1^, 50 mA g^−1^ 5139 mAh g^−1^, 100 mA g^−1^ 4098 mAh g^−1^, 200 mA g^−1^ 2581 mAh g^−1^, 500 mA g^−1^	–	60 cycles with the cut-off capacity of 1000 mAh g^−1^ at 100 mA g^−1^ 50 cycles with the cut-off capacity of 1000 mAh g^−1^ at 250 mA g^−1^ 20 cycles with the cut-off capacity of 1000 mAh g^−1^ at 500 mA g^−1^	[34]
PEO/LiTFSI/Li_7_La_3_Zr_1.4_Ta_0.6_O_12_	MCNTs	5 V	0.332 mS cm^−1^, 50 °C 1.01 mS cm^−1^, 70 °C	11,584 mAh g^−1^, 50 mA g^−1^ 10,300 mAh g^−1^, 100 mA g^−1^	1.15 V, 50 mA g^−1^	70 cycles with the cut-off capacity of 1000 mAh g^−1^ at 100 mA g^−1^	[139]
Li_1.4_Al_0.4_Ti_1.6_(PO_4_)_3_	Polyacrylonitrile (PAN)-derived CNFs	–	0.587 mS cm^−1^, RT	–	2.2 V, 50 mA g^−1^	50 cycles with the cut-off capacity of 500 mAh g^−1^ at 50 mA g^−1^	[140]
Li_1.4_Al_0.4_Ti_1.6_(PO_4_)_3_	MWCNT/Ru	–	0.92 mS cm^−1^, RT	6351 mAh g^−1^, 150 mA g^−1^	1.24 V, 50 mA g^−1^	50 cycles with the cut-off capacity of 500 mAh g^−1^ at 50 mA g^−1^	[141]
Li_1.4_Al_0.4_Ti_1.6_(PO_4_)_3_	MWCNTs	–	0.543 mS cm^−1^, RT	5255 mAh g^−1^, 60 mA g^−1^ 3479 mAh g^−1^, 80 mA g^−1^ 2519 mAh g^−1^, 100 mA g^−1^ 1794 mAh g^−1^, 120 mA g^−1^	∼2.01 V, 500 mAh g^−1^	50 cycles with the cut-off capacity of 600 mAh g^−1^ at 60 mA g^−1^	[142]
Li_1.5_Al_0.5_Ge_1.5_P_3_O_12_	Ru-based cathode	–	0.39 mS cm^−1^	–	0.6 V, 500 mA g^−1^ (150 °C)	980 cycles with the cut-off capacity of 500 mAh g^−1^ at 500 mA g^−1^ (150 °C) 150 cycles with the cut-off capacity of 500 mAh g^−1^ at 500 mA g^−1^ (150 °C)	[37]
Li_1.5_Al_0.5_Ge_1.5_(PO_4_)_3_	Ru/CNTs	–	0.7 mS cm^−1^, RT	4541 mAh g^−1^, 100 mA g^−1^	1.24 V, 100 mA g^−1^	45 cycles with the cut-off capacity of 500 mAh g^−1^ at 100 mA g^−1^	[143]
Ge-doped LiAlGeTi(PO_4_)_3_	MWCNT/Ru	–	1.04 mS cm^−1^, RT	–	1.4 V, 100 mA g^−1^	200 cycles with the cut-off capacity of 500 mAh g^−1^ at 100 mA g^−1^	[144]
Zn-doped Li_1.3_Al_0.3_Ti_1.7_(PO_4_)_3_	Fe3C/N-doped carbon tubes	5.75 V	2.45 mS cm^−1^, RT	19,244 mAh g^−1^, 50 mA g^−1^ 16,585 mAh g^−1^, 100 mA g^−1^	1.4 V, 100 mA g^−1^	180 cycles with the cut-off capacity of 500 mAh g^−1^ at 100 mA g^−1^	[145]
*Na–CO*_*2*_* batteries*							
NaClO_4_/TEGDME	Tetraethylene glycol dimethyl-treated MWCNTs (t-MWCNTs)	4.3 V	178 mS cm^−1^, RT	60,000 mAh g^−1^, 1 A g^−1^	~ 0.6 V, 1 A g^−1^	200 cycles with the cut-off capacity of 2000 mAh g^−1^ at 1 A g^−1^	[32]
Na(FSI)_27_(ClO_4_)_8_	Ru/C	–	~ 38 mS cm ^−1^, RT	148.1 mAh cm^−1^, 0.2 mA cm^−1^	0.64 V, 0.1 mA cm^−1^	1200 cycles at 0.1 mA cm^−1^	[146]
CNT/NaTFSI-IL gel electrolyte	Co-encapsulated N-doped carbon framework	4.2 V	5.3 mS cm^−1^	3094 mAh g^−1^, 0.1 mA cm^−2^ 1777 mAh g^−1^, 0.5 mA cm^−2^	1.7 V, 0.1 mA cm^−2^	367 cycles at 0.1 mA cm^−2^	[147]
PVDF-HFP/SiO_2_/NaClO_4_/TEGDME	Activated MCNTs		1 mS cm^−1^	5000 mAh g^−1^, 50 mA g^−1^	0.74 V, 100 mA g^−1^ 0.88 V, 200 mA g^−1^ 0.93 V, 300 mA g^−1^ 1.48 V, 500 mA g^−1^	400 cycles with the cut-off capacity of 1000 mAh g^−1^ at 500 mA g^−1^	[121]
Na_3.2_Zr_1.9_Ca_0.1_Si_2_PO_12_ /NaClO_4_/PVDF-HFP	Ru-ZIF-8	5.15 V	0.132 mS cm^−1^, RT	3320.7 mAh g^−1^, 100 mA g^−1^	–	70 cycles with a cut-off capacity of 500 mAh g^−1^ at 300 mA g^−1^	[148]
NaClO_4_/Na_3.2_Zr_1.9_Mg_0.1_Si_2_PO_12_ (NZM1SP)/PVDF-HFP	Ru/CNTs	4.7 V	NZM1SP: 1.16 mS cm^−1^, RT	7720 mAh g^−1^, 200 mA g^−1^	1.9 V, 200 mA g^−1^	120 cycles with the cut-off capacity of 500 mAh g^−1^ at 200 mA g^−1^	[149]
Na_2.7_Zr_2_Si_2_PO_11.7_F_0.3_-PVDF-HFP	Ru-CNTs	5.18 V	0.217 mS cm^−1^	6421.9 mAh g^−1^, 200 mA g^−1^	< 2 V, 200 mA g^−1^	234 cycles with the cut-off capacity of 500 mAh g^−1^ at 200 mA g^−1^	[150]
PVDF-HFP/NaClO_4_/SN	MWCNTs	4.86 V	1.32 mS cm^−1^, 50 °C	7624 mAh g^−1^, 50 mA g^−1^	1.54 V, 200 mA g^−1^ 2.08 V, 500 mA g^−1^	50 cycles with the cut-off capacity of 1000 mAh g^−1^ at 200 mA g^−1^	[151]
PEO/NaClO_4_/SiO_2_	MWCNTs	5.5 V	0.64 mS cm^−1^, 70 °C	800 mAh g^−1^, 50 mA g^−1^	–	240 cycles with the cut-off capacity of 500 mAh g^−1^ at 50 mA g^−1^	[35]
Na_3_Zr_2_Si_2_PO_12_	Ru/CNTs	–	0.8 mS cm^−1^, RT	28,830 mAh g^−1^, 100 mA g^−1^	1.4 V, 100 mA g^−1^	70 cycles with the cut-off capacity of 500 mAh g^−1^ at 50 mA g^−1^ 50 cycles with the cut-off capacity of 500 mAh g^−1^ at 100 mA g^−1^ 30 cycles with the cut-off capacity of 200 mAh g^−1^ at 100 mA g^−1^	[36]
Na_3_Zr_2_Si_2_PO_12_	Ru/MWCNTs	–	0.89 mS cm^−1^, RT	–	1.1 V, 100 mA g^−1^ 1.6 V, 200 mA g^−1^	105 cycles with the cut-off capacity of 500 mAh g^−1^ at 100 mA g^−1^ 68 cycles with the cut-off capacity of 500 mAh g^−1^ at 200 mA g^−1^	[152]

RT, room temperature

#### Desolvation Energy

In an electrolyte, alkali metal ions (Li^+^, Na^+^, K^+^) aggregate with solvent molecules through coordination bonds, hydrogen bonds and dipole interactions. Upon diffusing from the cathode to the anode, these ions need to undergo desolvation—the removal of coordinated solvent molecules—before combining with electrons for deposition. The solvation structure originates from the competitive coordination of solvents and anions with cations (Fig. 5d). The solvation of alkali metal ions affects their diffusion behavior in the electrolyte and the adsorption–desorption process at the interface and also changes the formation and structural characteristics of the SEI. If the desolvation process is difficult, the resulting polarization will affect the overall polarization of the battery [153, 154]. If the desolvation process is incomplete, it will damage the anode structure and even lead to the formation of metal dendrites, affecting the battery life [155].

The desolvation process of alkali metal ions is influenced by factors such as solvent type, additives and salt concentration. Changes in the electrolyte solvent molecules affect ion–solvent interactions, and the energy barrier of the desolvation process mainly depends on the strength of the association between ions and solvents. Reducing the desolvation barrier is expected to enhance the stability of metal deposition/stripping [156–159]. Through analyzing the interactions between metals and solvent/solute components, density functional theory (DFT) calculations can be employed to screen additives exhibiting stronger binding energies with ions. For instance, the introduction of LiPF_6_ into 1 M LiTFSI in TEGDME electrolyte demonstrates performance enhancement: The PF_6_^−^ anion displays higher binding energy with Li^+^ compared to TFSI^−^, effectively weakening Li^+^-TEGDME interactions and promoting the desolvation process. Furthermore, LiPF_6_ exhibits superior adsorption energy (1.21 eV) on the Li (001) surface versus LiTFSI (0.73 eV) [132]. The competitive decomposition with TFSI^−^ modifies interfacial chemistry, ultimately facilitating the formation of a LiF-rich SEI layer. Additives can change the solvation structure, thereby determining the behavior of solvents at the electrode interface. Adding ethylene sulfate (DTD) as an additive to the electrolyte, DTD can replace a certain proportion of solvents and participate in building the solvated shell layer of the central K^+^, thus changing the solvation structure [160]. Salt concentration can affect the stability of the electrolyte by regulating the number of free solvent molecules. As the proportion of LiTFSI salt and DMSO solvent gradually increases, the number of free DMSO molecules gradually decreases, while the amount of Li^+^–(DMSO)_4_ solvates increases. Solvates have higher activation energy barriers, effectively reducing solvent decomposition and improving stability [120, 161, 162].

### Interface Chemistry for Metal Anodes in Nonaqueous MCBs

Electrolytes and related interphases are at the core of battery chemistry [163]. These interphases are vital for preventing irreversible reactions with electrolytes, maintaining stable battery cycling and assisting in complex multiphase reactions.

#### Formation Mechanism of SEI

**Fig. 6:**
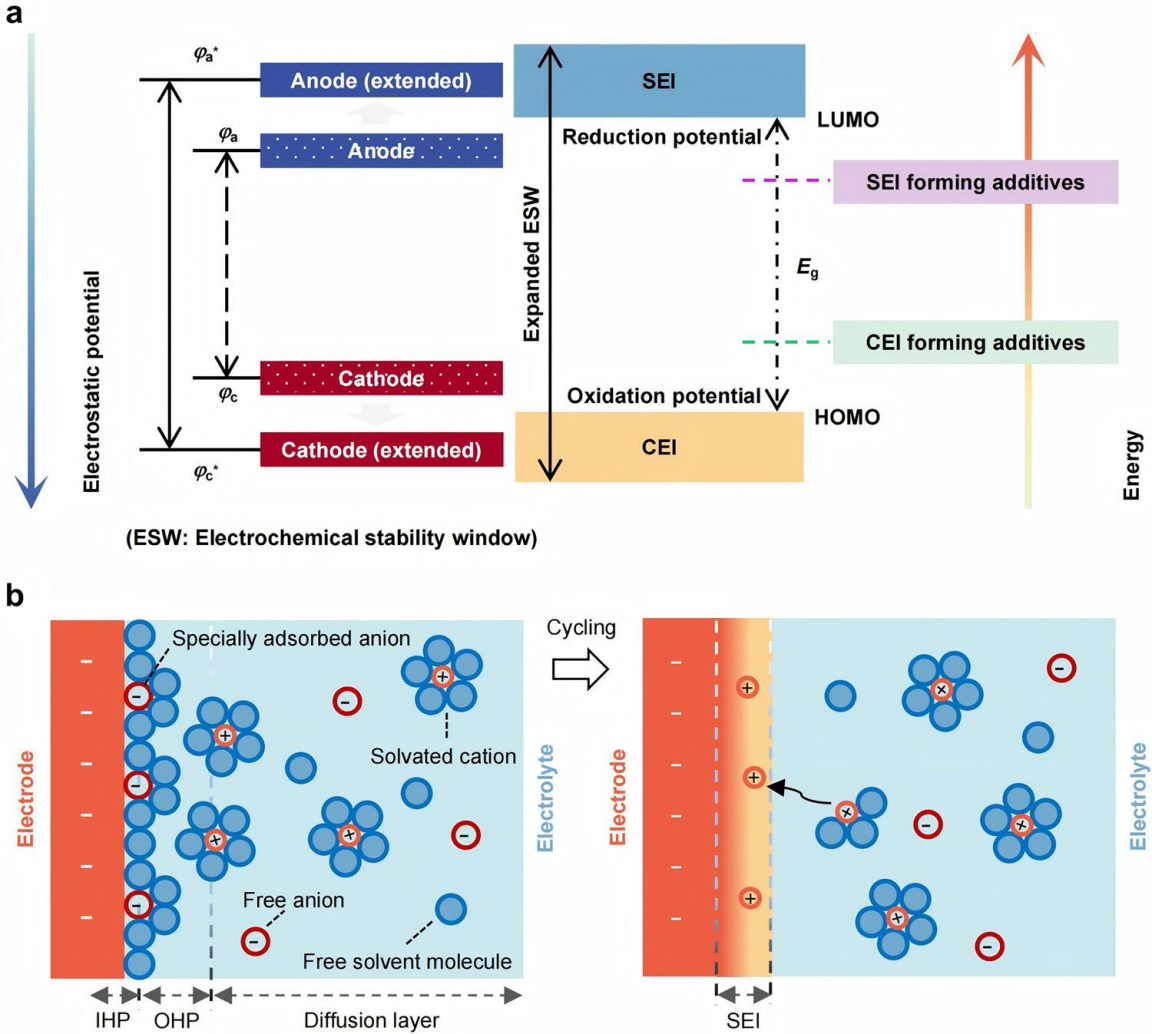
Formation mechanism of the SEI layer. **a** Schematic representation of energy states of the electrodes and electrolyte. **b** Schematic diagram of the EDL

During battery operation, the electrolyte will react with the electrodes in a complex multiphase reaction. Macroscopically, the SEIs are composed of the products formed by the reactions between the anode and the electrolyte during the initial charge–discharge cycle [164]. The formation of SEI is associated with the lowest unoccupied molecular orbital/highest occupied molecular orbital (LUMO/HOMO) of the electrolyte (Fig. 6a). When the LUMO of the electrolyte is lower than the Fermi level of the anode, electrons in the anode are transferred to the LUMO, leading to electrolyte reduction. Conversely, when the HOMO is higher than the Fermi level of the cathode, electrons will be transferred to the cathode, leading to electrolyte oxidation. During actual battery cycling, the reduction or oxidation of salts or solvents yields products that deposit on the electrode surface, forming a stable interface. This interface effectively widens the electrolyte's ESW beyond its intrinsic value while enabling ionic conduction [165, 166].

The generation of SEI in liquid batteries is related to the behavior of the electric double layer (EDL) [164, 167–169] and solvated coordination between electrodes and liquid electrolytes [154, 170]. Free solvent molecules and anions in the electrolyte are attracted or repelled by the electrode surface, forming an EDL on the nanointerface together with the charge on the electrode surface (Fig. 6b). This process precedes the reduction of the electrolyte and has an important influence on the electromotive force (EMF) of the battery and the reaction kinetics of SEI [171]. Due to the spatial effect of nanoscale, some anions and small neutral molecules adsorb on the inner Helmholtz plane (IHP), while large-sized solvated molecules adsorb on the outer Helmholtz plane (OHP). The electronic properties and microstructure of the electrode theoretically determine the specific adsorption of electrolyte components in IHP, as well as the initial structure and composition of SEI formed after the disappearance of such specific adsorption during the cycling process [172, 173]. The EDL is also closely related to the solvation structure of cations in the electrolyte. The solvation structure can be simply described as a sphere with two solvation shells—the cation located at the center, with an ordered “first solvation layer” consisting of continuous and uniform solvent molecules and a disordered “second solvation layer” composed of loosely arranged multiple molecules or ions [25, 174]. Anions distribute in this multi-solvation shell. Consequently, solvent molecules participating in solvation exhibit weakened adsorption on the electrode surfaces, leading to anion-induced interfacial phases. The solvation behavior is also influenced by the type and concentration of electrolytes [175].

Besides, in solid-state batteries, the physicochemical and mechanical properties of various solid components and the nature of solid–solid contacts all affect the formation of interfaces. These interfaces may include loose physical contacts, grain boundaries, chemical and electrochemical reactions, which will all increase the interface resistance.

#### Interfacial Issues for Metal Anodes

Alkali metals react significantly with organic liquid electrolytes [176]. The energy-level difference between the anode Fermi level and LUMO of the electrolyte determines the thermodynamic stability of the electrolyte on the anode side as well as the driving force for the formation of the SEI layer. The SEI layer of most nonaqueous metal anodes forms spontaneously below 1.0 V in organic liquid electrolytes [177, 178]. The SEI model was first proposed by Peled in 1979 [179], who believed that the SEI layer was a pure cation conductor. With the development of characterization techniques, the “double-layer model” has been proposed [180], which suggests that inorganic species have higher chemical stability toward metal anode than organic species, thus enriching the anode surface. Subsequently, the mosaic model inherited the hypothesis of the double-layer model, assuming that each component forms a pure microphase, and the SEI is an assembly of different microphases in a mosaic pattern [181–183]. Furthermore, the crystalline microphases are not concentrated on the surface of metal anode, which more accurately describes the complexity and dynamics of the SEI. The mosaic model has gradually been refined and widely accepted.

**Fig. 7:**
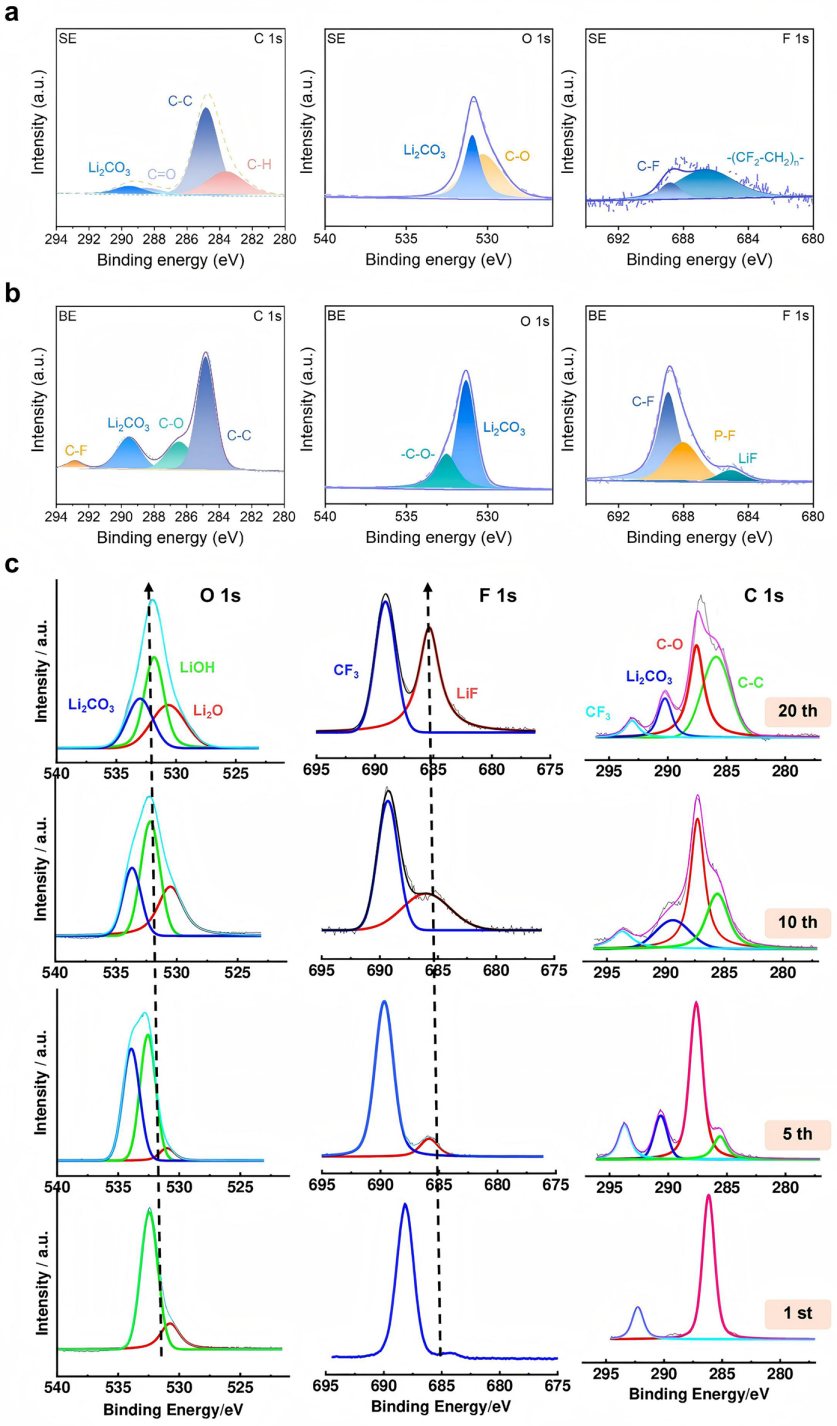
Characterization of the SEI composition on the Li surface after 100 cycles in the **a** single-salt electrolyte (SE) and **b** bissalt electrolyte (BE), reproduced with permission [132]. Copyright 2024, Elsevier. **c** XPS of the constituent elements of the compounds formed at the Li anode after different cycle numbers, reproduced with permission [186]. Copyright 2024, American Chemical Society

The SEI consists of composite electrolyte decomposition products. The organic components dominate ion transport, while the inorganic components confer passivation properties to the SEI [181, 184]. As mentioned above, the chemical composition and microstructure of the SEI undergo dynamic reconstruction during electrochemical cycling. To investigate this evolution, X-ray photoelectron spectroscopy (XPS) analysis was performed on lithium metal anodes after 100 cycles in 1 M LiTFSI/TEGDME electrolyte. The prominent presence of Li_2_CO_3_ (Fig. 7a) indicates preferential CO_2_ reduction at the lithium interface. Moreover, the formation of organic species through nucleophilic reactions between metallic lithium and the solvent was confirmed. The C–F bonding signals likely originate from rapid decomposition of TFSI^−^ anions into large fragments (e.g., Li_2_NSO_2_CF_3_, Li_*y*_C_2_F_*x*_ and CF_3_SO_2_Li) in baseline electrolytes [132, 185]. Upon introducing LiPF_6_ additive, the interfacial reaction kinetics are moderated through competitive adsorption, enabling the larger TFSI^−^ decomposition fragments to undergo further breakdown into smaller LiF molecules. This process leads to enhanced C–F signals and the emergence of distinct LiF peaks in XPS profiles (Fig. 7b). Comparative cycling studies further demonstrate dynamic SEI composition [186] (Fig. 7c). While Li_2_CO_3_ dominates the first-cycle spectrum, the intensity markedly diminishes by the fifth cycle. In contrast, LiOH maintains prominence throughout all cycles, emerging as the predominant phase during prolonged cycling. Additional spectral features confirm CF_3_ and LiF as persistent SEI constituents.

Ideally, the SEI layer can prevent the continuous decomposition of the electrolyte on the metal surface while enabling uniform ion flux distribution [187, 188]. Actually, the SEI layer is uneven, and during the process of ion plating/stripping, significant volume changes easily lead to cracks in the SEI [189, 190]. Due to the low interfacial resistance of the exposed metal surface, ions tend to deposit preferentially at the cracks, forming dendrites [188, 191]. The newly deposited metal is highly reactive with the electrolyte and reacts to form a new SEI. Subsequently, during the stripping process, the roots of dendrites preferentially receive electrons and dissolve, and the stripped metal is wrapped by SEI and loses electrochemical activity [192–194]. During long-term cycling, repeated damage and reconstruction of the SEI layer lead to continuous consumption of both the electrolyte and the metal, resulting in increased interface resistance. The gradual accumulation of stripped metal can also lead to internal short circuits in the battery. Adjusting the composition or the additives of electrolytes can help to protect the anode from side reactions. For example, PF_6_^−^ can induce the in situ construction of a stable mixed SEI layer (Li_2_CO_3_/LiF-rich), inhibiting the continuous side reactions of Li, electrolyte and CO_2_, and extending the long-term durability of the anode [132, 134, 137]. Br^−^ can promote the decomposition of Li_2_CO_3_ ($2{\text{Li}}_{2}{\text{CO}}_{3}+\text{C}+2{\text{Br}}_{2}\to 4\text{LiBr}+3{\text{CO}}_{2}$) [111]. Mn phthalocyanine (MnPc) can act as an electrolyte catalyst, mediating charge transfer during discharging by binding to electrochemically reduced radical anions, thereby promoting the formation and reversible release of Li_2_CO_3_ and C products [195].

While replacing organic electrolytes with solid-state electrolytes may help inhibit continuous reactions between the electrolyte and metal, and solid-state electrolytes are a promising candidate to suppress uneven electrodeposition of metals and/or hinder the formation of dendrites [196, 197]. On the one hand, the narrow ESW of most solid-state electrolytes makes them possible to be decomposed during battery operation. On the other hand, it is prone to forming voids at the electrode–electrolyte interface due to the significant volume changes caused by multiple depositions and extractions of the anode, even under lower current densities [198, 199]. In addition, the properties of the SEI layer are related to its electronic and ionic conductivities. When the SEI exhibits high ionic conductivity and low electronic conductivity, the formed SEI is the most stable, avoiding the continuous consumption of solid-state electrolytes.

#### Advances of SEI Chemistry in Nonaqueous MCBs

An ideal SEI requires several essential characteristics, such as intrinsic stability, dense morphology, high ionic conductivity, and good mechanical integrity [200–203]. However, the formation of SEI is a spontaneous chemical reaction that is hard to precisely control. Currently, the modulation of interfacial chemistry is achieved through the structure and morphology of the electrode as well as the composition of the electrolyte [174]. Anode failure, dissolved CO_2_ [204], diffused RM molecules [205] or by-products from electrolyte decomposition, can be limiting factors in the cycling performance of MCBs. They lead to dendrites and surface passivation, which cause metal pulverization and lead to cell failure [204, 206, 207].

Earlier studies have suggested that the main discharge product of MCBs is carbonate, and the presence of CO_2_ can lead to the formation of stable carbonate/C protective coatings on nonaqueous metal surfaces [31, 208]. The charge-transfer resistance of the passivated anode continues to increase with cycling, which implies that the anode polarization accounts for an increasing share of the overall cell polarization during the operation of MCBs. By adding an appropriate mediator to the electrolyte, a SEI film can be constructed at the electrode surface to maintain the stability of the electrode structure. For example, charge–discharge passivation of K metal in a TEGDME-based electrolyte containing 2.7 M KFSI results in the formation of an artificial SEI film on its surface enriched with KF and carboxylate/carbonyl species [91]. This well-designed KF protective layer is a fast conductor of K^+^ and homogenizes the nucleation sites, which inhibits to some extent the formation of irreversible K during the plating process. It helps to stabilize the K anode and reduce polarization. Although some studies have attributed the failure of metal anodes in part to CO_2_, CO_2_ has also been recognized as a gaseous additive for the protection of nonaqueous metals [204].

Constructing an artificial SEI for metal anode is an effective strategy to improve the interfacial compatibility. A compatible Na/SN-based electrolyte interface can be formed by introducing a NaF-rich compact phase on the surface of Na through the chemical reaction between fluoroethylene carbonate (FEC)-Na^+^ and Na metal [151]. Compared with common organics, fluorinated organics have unique physicochemical properties, hydrophobicity and high oxidative stability [209, 210]. The in situ formed NaF-rich intermediate phase not only prevents side reactions between the SN-based electrolyte and the anode but also regulates the uniform deposition of dendrite-free Na. There is effective improvement of electrolyte–electrode interfacial contact during the construction of PSEs via in situ polymerization [211, 212]. Thanks to the in situ reaction, the GPE can form tight interfaces with both the cathode and anode, respectively. For example, GPEs were constructed by in situ polymerization in the framework of a mixture of the fluoropolymers trifluoroethyl methacrylate (TFMA) and polyethylene glycol diacrylate (PEGDA) [137]. TFMA is preferentially reduced on the lithium anode and participate in the formation of SEI. The active − CF_3_ groups were introduced to promote the formation of a fish scale-like LiF-rich SEI layer on the anode. LiF has high surface energy, low Li^+^ diffusion resistance and excellent electronic insulating properties, which induce uniform and dense deposition of lithium and inhibit the deep lithium layer from being further etched [213–216]. Also, the participation of organic components is conducive to optimizing the SEI and withstanding the volume change of the anode during repeated cycling, effectively suppressing the chalking of electrodes [217, 218].

## Summary and Outlook

**Fig. 8:**
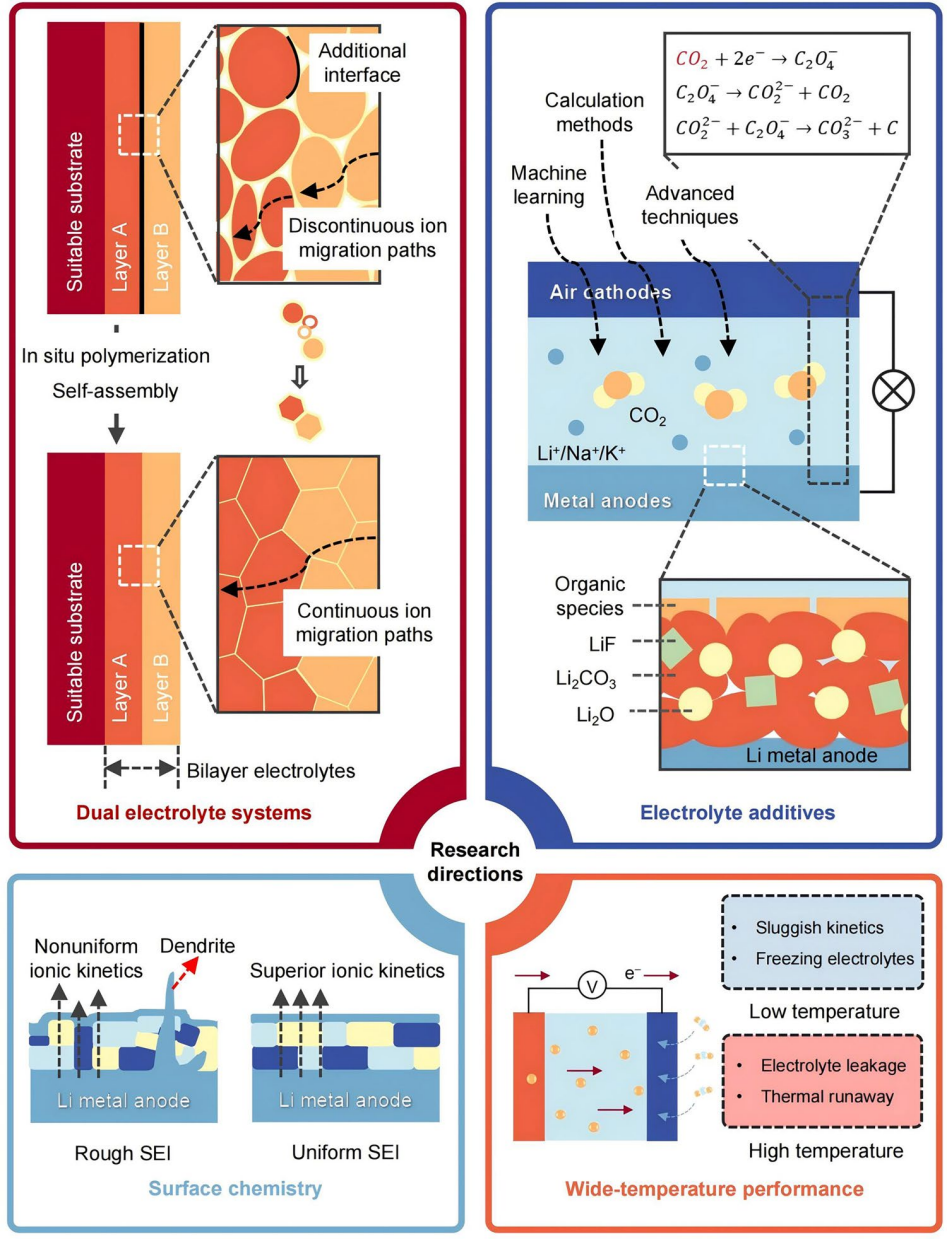
Future perspectives on the research direction of electrolytes and interface engineering for nonaqueous MCBs

Nonaqueous MCBs are a relatively new and developing research system to date; there is limited understanding of their electrochemical mechanisms. This review discussed the research progress of MCBs, and the chemical and electrochemical mechanisms of batteries and electrolytes were explored to better understand how these relate to the optimization of electrolytes and interface engineering. Although great progress has been made in recent years in electrolytes of nonaqueous MCBs and interface engineering, there are still many challenges that need to be further explored. Here, we propose several potential research directions (Fig. 8):

1. Designing a dual-electrolyte system. PSEs have a narrow ESW, and constructing bilayer PSEs is expected to solve this problem. However, the bilayer structure leads to additional electrolyte/electrolyte interfacial resistance and discontinuous ion migration paths [219, 220]. It is necessary to find a suitable substrate as well as to design a rational structure.
2. Selecting the matched electrolyte additives. Machine learning and calculation methods enable efficient screening of effective additives, providing mechanistic insights into battery electrochemical reactions [221] through advanced techniques, thereby facilitating a high-performance nonaqueous MCB [222].
3. Regulating surface chemistry and dynamics. Ion conversion in the electrolyte and the transfer process through the electrolyte/electrode interface can affect the cycle life of the cell. Continuing to improve the compatibility of electrodes and electrolytes, especially solid-state electrolytes, is an important interface engineering strategy.
4. Enhancing the performance of nonaqueous MCBs over wide-temperature ranges, variations in ambient temperature critically influence the performance of nonaqueous MCBs and nonaqueous MCBs. As a core component, electrolytes govern the batteries’ behavior under extreme thermal conditions [223]. At low temperatures, the reduced ionic conductivity of electrolytes severely restricts the transport kinetics of Li^+^/Na^+^/K^+^ ions and CO_2_, while the increased desolvation energy barrier at the electrode/electrolyte interface leads to elevated charge-transfer impedance and overpotential [20]. Novel electrolyte formulations and structural designs are imperative to improve the discharge voltage and enhance the mass transfer rate, to enable reliable operation under harsh thermal conditions [224]. For liquid electrolytes, at low temperatures, systems with low desolvation energy [225, 226], low melting points and superior low-temperature ionic conductivity are essential. The electrolyte concentration also significantly impacts the low-temperature performance. Low-concentration electrolytes permit free ion mobility but suffer from insufficient transference number. In high-concentration electrolytes, anions inevitably appear in the primary solvation sheath layer of Li^+^/Na^+^/K^+^ ions, which predominantly govern the formation of SEI, thereby stabilizing the electrode–electrolyte interfaces [225, 227, 228]. However, the high viscosity impedes practical low-temperature operation [229]. Introducing low-viscosity co-solvents can mitigate the concentration polarization of the electrolyte and improves ionic conductivity, thereby boosting discharge capacity. Local high-concentration electrolytes further address this challenge by incorporating low-polarity diluents, which preserve the benefits of the high salt concentration while reducing viscosity and maintaining high ion mobility [121, 130]. The failure of batteries at high temperatures primarily stems from compromised electrochemical stability, interfacial degradation and thermal instability of electrolytes [230]. Although high temperatures enhance ionic conductivity, they accelerate electrolyte decomposition and parasitic side reactions. Enhancing flame retardancy is crucial to mitigate volatility risks in liquid electrolytes, strategies such as electrode surface modification or additives that form stable SEI layers can reinforce interfacial stability. Furthermore, solid-state electrolytes, with their high Young’s modulus (effectively suppressing dendrite growth) and temperature-insensitive ionic conductivity (minimizing solvent decomposition risks at high temperatures), offer a promising pathway for developing wide-temperature-tolerant nonaqueous MCBs.

Overall, although MCBs have shown great advantages, challenges remain before their practical applications. In the future, novel material design and advanced characterization should be combined, and if excellent cycling performance and large-scale production can be widely achieved, MCBs will be promising for energy transition and greenhouse gas emission reduction.
